# Retraction: Colon cancer-induced interleukin-35 inhibits beta-catenin-mediated pro-oncogenic activity

**DOI:** 10.18632/oncotarget.28325

**Published:** 2022-12-17

**Authors:** Yueqiang Jiang, Yanling Ma, RuiChao Li, JianHai Sun

**Affiliations:** ^1^Department of Gerontology, Tongji Hospital, Tongji Medical College, Huazhong University of Science and Technology, Wuhan, Hubei Province, China; ^2^Department of Oncology, the Third People’s Hospital, Wuhan, Hubei Province, China; ^*^Co-first author

**This article has been retracted:** During its investigation, Oncotarget requested the original data for this paper from the authors. The authors state: “After investigation, we determined that some original data were lost and we cannot repeat the results, making data interpretation difficult and conclusion unreliable.” All authors have therefore agreed to the retraction of this paper from Oncotarget.

Original article: Oncotarget. 2018; 9:11989–11998. 11989-11998. https://doi.org/10.18632/oncotarget.22857

